# Understanding Esophageal Diverticular Bleeding: Causes, Symptoms, and Treatment

**DOI:** 10.7759/cureus.42991

**Published:** 2023-08-05

**Authors:** Abeer Qasim, Priscilla Lajara, Abhilasha Jyala, Harish Patel, Haider Ghazanfar

**Affiliations:** 1 Internal Medicine, BronxCare Health System, Bronx, USA; 2 Medicine/Gastroenterology, BronxCare Health System, Bronx, USA

**Keywords:** hematemesis, diverticulectomy, esophagogastroduodenoscopy, halitosis, esophageal diverticulum

## Abstract

Esophageal diverticulum refers to a sac or protrusion formed by the epithelial-lined tissue of the esophagus. It can exist as either a true diverticulum involving all layers of the esophagus or a false diverticulum. Most cases of esophageal diverticula are acquired conditions that primarily affect older individuals. Typically, patients with a known motility disorder experience dysphagia as a common symptom of esophageal diverticulum; other symptoms include recurrent pneumonia, hoarseness, and halitosis. Failure to diagnose this condition promptly or accurately can result in more severe complications, such as gastrointestinal bleeding, lung abscesses, aspiration pneumonia, and cancer development. In this report, we present the case of a 68-year-old female who arrived at the emergency department with symptoms of epigastric discomfort and hematemesis and was found to have diverticula in the middle portion of the esophagus.

## Introduction

Esophageal diverticulum is more prevalent in the male gender and between the ages of 60 and 70 years. Esophageal diverticulum tends to occur more commonly at the gastroesophageal junction [1]. The estimated incidence of esophageal diverticula is one per 500,000 people per year, whereas the prevalence is 0.06%-4% based on radiology and endoscopic findings [2]. An esophageal diverticulum can be categorized according to the site of occurrence, including Zenker's diverticulum (pharyngoesophageal), typically found in the rear part of the throat, slightly above the esophagus. Mid-esophageal diverticula are positioned in the middle of the chest, and epiphrenic diverticula are located above the diaphragm. The common symptoms of this condition include dysphagia, odynophagia, regurgitation, and epigastric pain. However, some patients may be asymptomatic [3]. We present a case of a 68-year-old female who presented with a complaint of epigastric pain and hematemesis.

## Case presentation

A 68-year-old female arrived at the emergency department with a complaint of vomiting, weakness, and epigastric discomfort for the past day. The vomiting was initially nonbilious and non-bloody but later on had two episodes of hematemesis. The patient's past medical history was significant for human immunodeficiency virus (HIV) infection, chronic kidney disease (CKD), chronic obstructive pulmonary disease (COPD), hypertension, hepatitis C infection, liver cirrhosis, and polysubstance abuse. The patient's past surgical history was significant for cholecystectomy. There was no family history of stomach and colon cancers. The patient has no known allergies. She is an active smoker, uses heroin and cocaine on a daily basis, and drinks two glasses of alcohol once a week. The physical examination was unremarkable. Her initial laboratory findings are presented in Table 1.

**Table 1:** Laboratory results at the time of admission Hgb, hemoglobin; MCV, mean corpuscular volume; MCH, mean corpuscular hemoglobin; MCHC, mean corpuscular hemoglobin concentration; MPV, mean platelet volume; RDW, red cell distribution width

Laboratory Test	Result	Normal Range
WBC Count	11.4 k/μL	4.8-10.8 k/μL
RBC Count	3.70 mil/μL	4.00-5.20 mil/μL
Hgb	9.1 g/dL	12.0-16.0 g/dL
Hematocrit, Whole Blood	28.7%	42.0%-51.0%
MCV	77.5 fL	80.0-96.0 fL
MCH	24.5 pg	27.0-33.0 pg
MCHC	31.6 g/dL	33.0-36.0 g/dL
MPV	8.7 fL	8.0-12.0 fL
RDW	16.3%	10.5%-14.5%
Platelet	189 k/μL	150-400 k/μL
Lactic Acid Level	0.5 mmoles/L	0.5-1.6 mmoles/L
Blood Urea Nitrogen, Serum	29.0 mg/dL	6.0-20.0 mg/dL
Creatinine, Serum	2.6 mg/dL	0.5-1.5 mg/dL
Albumin, Serum	3.9 g/dL	3.2-4.6 g/dL
Bilirubin, Serum Total	0.3 mg/dL	0.2-1.1 mg/dL
Bilirubin, Serum Direct	<0.2 mg/dL	0.0-0.3 mg/dL
Alkaline Phosphatase, Serum	101 U/L	43-160 U/L
Aspartate Transaminase, Serum	15 U/L	9-36 U/L
Alanine Aminotransferase, Serum	7 U/L	5-40 U/L
Lipase, Serum	38 U/L	<61 U/L

**Figure 1:**
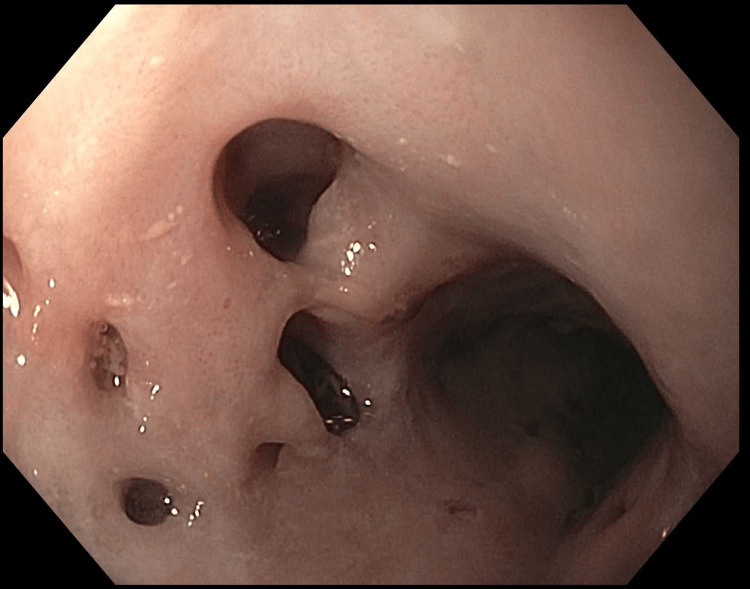
Diverticula in the middle segment of the esophagus containing blood clots

The patient underwent esophagogastroduodenoscopy (EGD), which was remarkable for the presence of diverticula in the middle segment of the esophagus containing blood clots (Figure 1).

No active ongoing bleeding was observed. She did not have repeat episodes of hematemesis. She was started on oral omeprazole 40 mg once a day, 30 minutes before breakfast, and was advised to avoid nonsteroidal anti-inflammatory drugs (NSAIDs). The patient's chest CT with contrast was unremarkable. Her clinical condition improved, and she was discharged. During her visit to the gastroenterology clinic, she had no complaints, and all her symptoms have been resolved. The laboratory results showed a stable hemoglobin level.

## Discussion

Esophagoscopy and barium esophagogram are the imaging modalities that can help in visualizing and assessing the diverticulum's location and size and any potential complications associated with the diverticula. Esophagogastroduodenoscopy (EGD) needs to be done to confirm the diagnosis of esophageal diverticulum.

Patients with mid-esophageal and epiphrenic diverticula who do not have symptoms do not require treatment. Surgical and endoscopic interventions are reserved for patients with symptoms [4]. Endoscopy has a limited role, whereas surgery represents the established medical approach for individuals experiencing severe respiratory or debilitating symptoms linked to an epiphrenic diverticulum, with myotomy being the fundamental surgical procedure. At times, it becomes necessary to target the diverticulum with specialized treatments such as diverticulectomy or diverticulopexy.

More than 40% of esophageal diverticula are asymptomatic and are incidentally discovered during endoscopy [5]. Most symptomatic patients typically experience mild intermittent dysphagia. Some patients can present with regurgitation when changing position, excessive salivation, odynophagia, and severe epigastric pain and, in rare cases, can present with life-threatening upper gastrointestinal bleeding [6,7].

**Figure 2:**
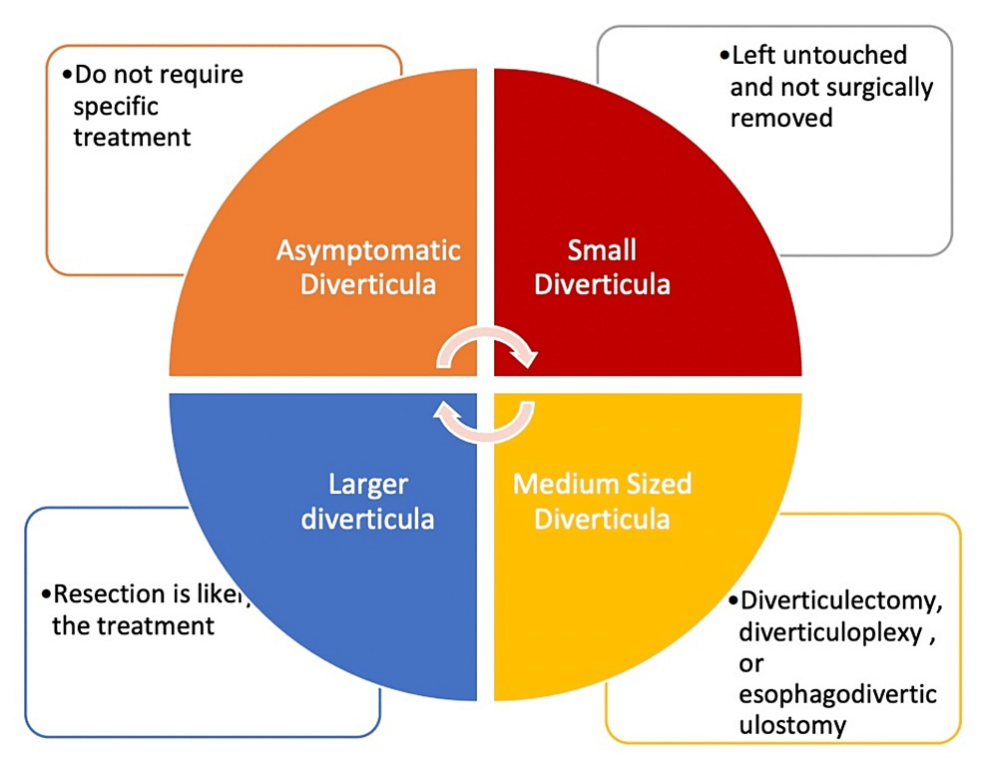
Various treatment options depending on the size of esophageal diverticula Small-sized diverticula is defined as <1 cm. Medium-sized diverticula is 1-3 cm. Large-sized diverticula is >4 cm. Figure drawn by Dr. Abeer Qasim

The management of esophageal diverticula should be determined based on the underlying pathophysiology. The treatment approach varies depending on different scenarios, as shown in Figure 2 [8].

In cases where patients experience severe symptoms such as dysphagia particularly with solids, regurgitation of undigested food, chronic cough or aspiration due to food getting into the airways, weight loss or malnutrition due to difficulty eating and recurrent infections such as pneumonia, or upper gastrointestinal bleeding, surgical and endoscopic interventions are recommended to offer long-term relief from these symptoms [9]. Treatment options may involve endoscopic myotomy, diverticulectomy, diverticulopexy, or diverticular inversion. However, in the case of our patient, who presented with upper gastrointestinal bleeding, improvement was observed through medical management.

In the case of patients with motility disorders, the alternative procedures of choice may include pneumatic dilation and botulinum toxin injections [10]. Therapeutic interventions are not advised in asymptomatic patients as the likelihood of disease progression is low [7,11]. However, the management of asymptomatic cases remains a subject of debate due to studies suggesting the occurrence of aspiration (45%) and potentially life-threatening pulmonary complications (15%) in certain patients [12]. Other postoperative interventions include esophageal leak, dysphagia, and acid reflux [13].

Failure to promptly diagnose and treat esophageal diverticulum can lead to the development of complications. While uncommon, these complications can be severe and encompass esophageal bleeding, obstruction, perforation, vocal cord paralysis, and fistula formation [14,15].

The patient's age, the size of the pouch, and the duration of the injury are the main factors for the increased risk of malignancy, especially squamous cell carcinoma [14]. Carcinogenesis may be caused by chronic irritation by food, inflammation, and repeated injury [14], and it is often advanced at the time of diagnosis.

Esophageal diverticula have an estimated incidence of 1:500,000 per year and a mortality rate of 7%-14% [16]. The occurrence of mortality is frequently associated with both abnormal motility and esophageal leak postoperatively [9]. Overall, the prognosis of esophageal diverticula is generally favorable with appropriate management and regular monitoring. It is important to address any associated complications promptly to ensure optimal outcomes.

## Conclusions

In conclusion, esophageal diverticulum presents a diverse spectrum of clinical scenarios that necessitate a comprehensive and multidisciplinary diagnostic and management approach. Moreover, patients presenting with severe symptoms, including hematemesis, involve a meticulous and systematic approach to evaluating the potential underlying causes of this alarming symptom. Medical professionals must consider various factors, including the patient's medical history, presenting symptoms, physical examinations, and diagnostic tests. By distinguishing between various possible sources of gastrointestinal bleeding, such as peptic ulcers, esophageal varices, Mallory-Weiss tears, or malignancies, clinicians can accurately pinpoint the root cause and provide timely and effective interventions. Physicians also need to be aware of the possibility of esophageal diverticulum presenting as hematemesis, as blindly advancing the scope into the diverticulum can lead to esophageal perforation. Collaborative efforts between gastroenterologists, surgeons, and other healthcare professionals are essential in providing comprehensive care to individuals with esophageal diverticula.
